# Antibiotic use in Chilean salmon aquaculture: antimicrobial resistance, sustainability, and One Health implications

**DOI:** 10.3389/fmicb.2026.1810226

**Published:** 2026-05-08

**Authors:** Karla Camacho-Méndez, Lina J. Cortés, Juan Parás-Silva, Sebastián Higuera-Llantén, Richard Covarrubia-López, Diego Lira-Velásquez, Felipe Vásquez-Ponce, Manuel Alcalde-Rico, Fernando O. Mardones, Jorge Olivares-Pacheco

**Affiliations:** 1 Escuela de Medicina Veterinaria, Facultad de Recursos Naturales y Medicina Veterinaria, Universidad Santo Tomás, Talca, Chile; 2 Instituto de Ciencias e Innovación en Medicina, Facultad de Medicina, Clínica Alemana Universidad del Desarrollo, Santiago, Chile; 3 Grupo de Resistencia Antimicrobiana en Bacterias Patógenas y Ambientales (GRABPA), Instituto de Biología, Pontificia Universidad Católica de Valparaíso, Valparaíso, Chile; 4 NeoSensing SpA, Santiago, Chile; 5 Instituto de Biomedicina de Sevilla (IBiS), Hospital Universitario Virgen Macarena, CSIC, Universidad de Sevilla, Sevilla, Spain; 6 CIBER de Enfermedades Infecciosas, Instituto de Salud Carlos III, Madrid, Spain; 7 Division of Global Agriculture and Food Systems, Royal (Dick) School of Veterinary Studies, University of Edinburgh, Edinburgh, United Kingdom

**Keywords:** antimicrobial stewardship, metaphylactic antibiotic use, *Piscirickettsia salmonis*, salmon aquaculture resistome, salmonid aquaculture

## Abstract

Aquaculture has expanded rapidly over recent decades, positioning salmon farming as a major contributor to global food security while intensifying concern over antimicrobial use and the emergence of antimicrobial resistance (AMR). Chile, the world’s second-largest producer of farmed salmon, represents a critical case study because of its historically high dependence on antibiotics, particularly florfenicol and oxytetracycline. This dependence is driven largely by the endemic burden of salmon rickettsial syndrome (SRS), caused by *Piscirickettsia salmonis*, for which currently available vaccines have shown limited and inconsistent effectiveness under commercial farming conditions. In this review, we examine antimicrobial use patterns, resistance dynamics, environmental dissemination, and regulatory frameworks associated with Chilean salmon aquaculture within a One Health perspective. We show how intensive production systems, persistent disease pressure, and operational constraints have favored a predominance of metaphylactic treatments delivered through medicated feed. Although operationally feasible, this strategy entails major biological and ecological drawbacks, including heterogeneous drug exposure, unnecessary treatment of clinically healthy fish, and sustained selective pressure on microbial communities associated with fish, sediments, and surrounding aquatic environments. We further argue that antimicrobial dependence in Chilean salmon aquaculture is sustained by multiscale drivers that extend beyond pathogen burden alone. These include the structure of an export-oriented production model, the mismatch between long production cycles and prolonged disease susceptibility, incomplete incorporation of host resistance into preventive strategies, and the destabilizing effects of environmental stressors such as harmful algal blooms and low-oxygen conditions. Although acquired resistance in major salmon pathogens remains limited and, in some cases, mechanistically unresolved, aquaculture-associated microbiota constitute important reservoirs of antibiotic-resistant bacteria and resistance genes. Mobile genetic elements linked to aquaculture environments have also been detected in opportunistic and clinically relevant human pathogens, highlighting ecological connectivity and broader public health relevance beyond farm boundaries. We conclude that reducing antibiotic dependence in Chilean salmon aquaculture will require a transition toward preventive, biologically informed, and data-driven health management, supported by improved vaccine performance against SRS, integrated genomic and environmental surveillance, and regulatory thresholds grounded in robust biological evidence.

## 1 Global aquaculture and antibiotic use

Aquaculture surpassed capture fisheries in 2022, accounting for 51% of global aquatic animal production (FAO, 2024). This rapid expansion, driven by intensification and the growth of international trade, has strengthened the industry but has also increased physiological stress in farmed animals, making them more susceptible to infectious outbreaks and leading to substantial economic losses (Blanchard et al., 2017; Araujo et al., 2022). Intensification is closely associated with a higher incidence of bacterial diseases and a growing reliance on antibiotics, mainly administered through medicated feed in intensive production systems. These compounds are used for therapeutic, prophylactic, and metaphylactic purposes; however, indiscriminate application reduces clinical efficacy, disrupts aquatic microbial communities, and promotes opportunistic infections, a trend recognized for several decades (Petersen et al., 2002; Santos and Ramos, 2018; Ferri et al., 2022; Bondad-Reantaso et al., 2023; Asif et al., 2024). Global estimates highlight the scale of antimicrobial use in aquaculture: consumption reached 10,259 tons in 2017 and is projected to increase by 33%, reaching 13,600 tons by 2030 (Schar et al., 2020). The Asia-Pacific region accounts for 93.8% of this use, with China alone representing 57.9% (Schar et al., 2020). This heavy dependence on antimicrobials exacerbates the emergence and spread of antimicrobial resistance (Mo et al., 2017; Miranda et al., 2018: Milijasevic et al., 2024). Antibiotics are used across a wide range of cultured species, including shrimp, tilapia, carp, catfish, and salmonids, with tetracyclines, sulfonamides, and phenicols being the most widely used classes (Milijasevic et al., 2024). Although antibiotics remain essential for disease control, their intensive use drives resistance development and disrupts aquatic ecosystems (Farías et al., 2024; Mohammed et al., 2025).

From a One Health perspective, antibiotic-resistant bacteria (ARBs) and antibiotic resistance genes (ARGs) originating from aquaculture systems may be transferred to humans and terrestrial animals through the food chain, shared aquatic environments, or horizontal gene transfer (Okeke et al., 2022; Milijasevic et al., 2024). The absence of harmonized global regulatory frameworks further complicates the implementation of effective antimicrobial stewardship strategies in aquaculture (Gadhiya et al., 2025).

## 2 Salmon farming worldwide and in Chile

Salmon farming represents one of the most dynamic and technologically advanced sectors within global aquaculture, combining high-value production with intensive husbandry practices (Cojocaru et al., 2024; Stige et al., 2024; Cantillo and Deshpande, 2025). Production systems are characterized by high stocking densities, specialized feeds, and sophisticated health management strategies, closely resembling intensive terrestrial animal farming (Henriksson et al., 2015; Cabello and Godfrey, 2023). In 2023, global salmonid aquaculture, dominated by Atlantic salmon (*Salmo salar*), rainbow trout (*Oncorhynchus mykiss*), and coho salmon (*Oncorhynchus kisutch*), was largely concentrated in Norway (1,632,502 tons), Chile (1,089,641 tons), the United Kingdom (164,529 tons), and Canada (82,729 tons) (FAO, 2025). Despite comparable production volumes, salmon-producing countries display strikingly different patterns of antimicrobial use, reflecting divergent disease-management strategies, regulatory frameworks, and vaccination coverage. In Norway and other European producers, large-scale vaccination programs combined with stringent regulations have reduced antibiotic use by more than 99%, reaching levels below 1 g of active ingredient per ton of salmon produced (Lillehaug et al., 2018; Hoseinifar et al., 2024). Consistent with this approach, only 16 veterinary antibiotic prescriptions were issued across all Norwegian salmon farms in 2019 (NORM/NORM-VET 2019, 2019).

**Figure 1:**
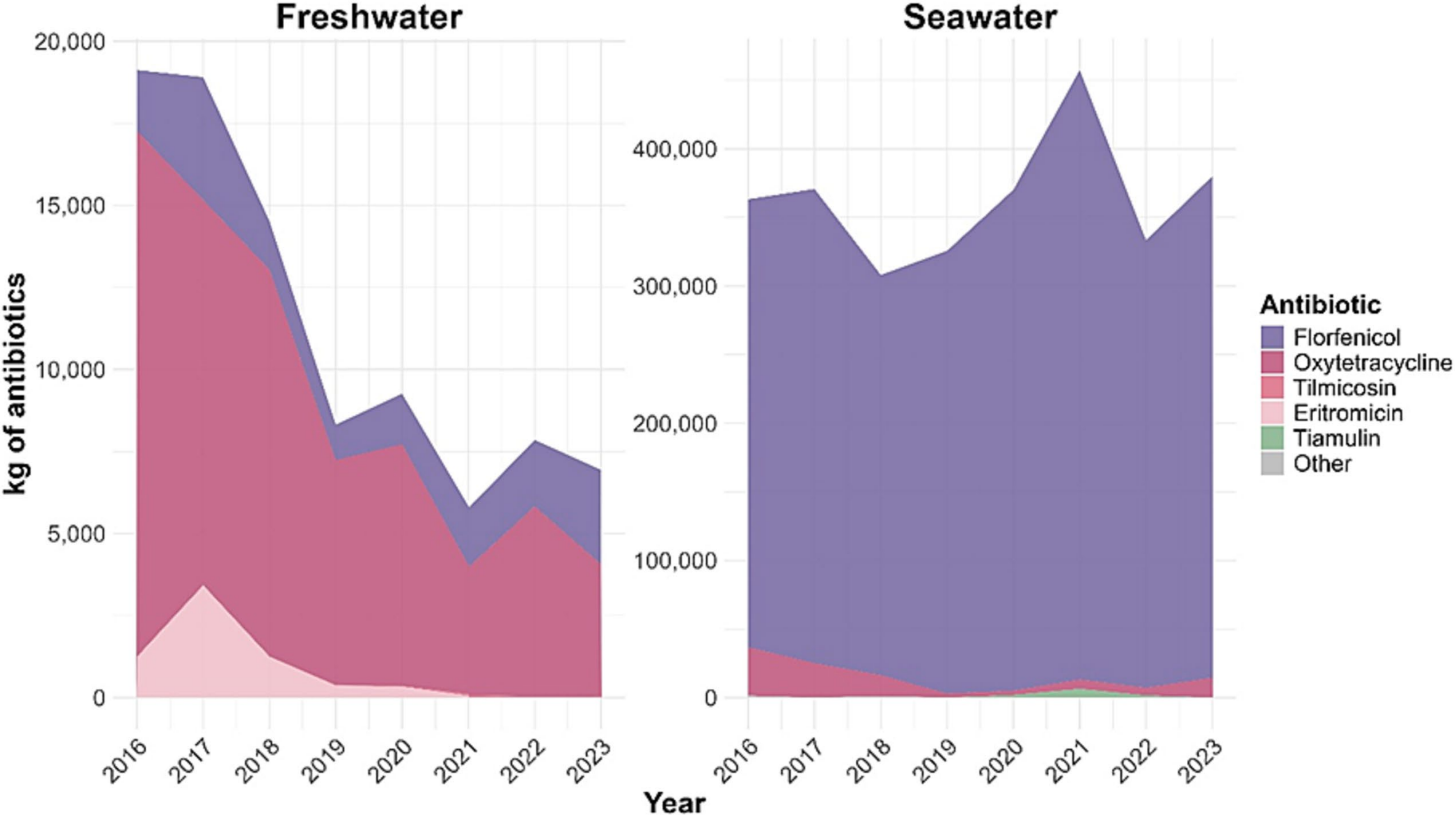
Annual antimicrobial use in Chilean salmon aquaculture by production phase and antibiotic class (2016–2023). Stacked area plots illustrate the total kilograms of active ingredient used annually during the freshwater and seawater production phases. Florfenicol (purple) and oxytetracycline (red) were the predominant antimicrobials throughout the study period. Florfenicol consistently dominated use during the seawater grow-out phase, whereas oxytetracycline showed comparatively greater use during the freshwater phase, particularly in 2020.

In contrast, Chile remains the world’s largest consumer of antibiotics in salmon aquaculture, with annual use exceeding 300–500 tons, predominantly florfenicol and oxytetracycline (SERNAPESCA, 2025) (Figure 1). This high consumption is largely driven by the persistent prevalence of *Piscirickettsia salmonis*, the etiological agent of salmon rickettsial syndrome (SRS), which continues to pose major challenges to fish health management (Miranda et al., 2018; Hillman et al., 2020; SERNAPESCA, 2025). By comparison, Canada and the United Kingdom report relatively low antimicrobial use, supported by effective vaccination strategies, strong biosecurity measures, and robust regulatory oversight (Morrison and Saksida, 2013; Ojasanya et al., 2022; Hoseinifar et al., 2024; Jonah et al., 2024).

These international contrasts clearly illustrate how vaccination coverage, farm management practices, and regulatory enforcement shape antimicrobial consumption patterns in salmon aquaculture. In the Chilean context, the sustained reliance on antibiotics not only challenges the long-term sustainability of the industry but also increases environmental contamination and heightens the risk of antimicrobial resistance dissemination into marine ecosystems, with potential repercussions for global public health (Cabello, 2006; FAO, 2024).

### 2.1 Vaccination strategies and limitations in salmon aquaculture

Vaccination is widely recognized as the most effective preventive strategy for controlling infectious diseases in aquaculture and for reducing reliance on antimicrobials (Assefa and Abunna, 2018). In teleost fish, vaccines have been successfully implemented against a range of viral and bacterial pathogens and have contributed to substantial reductions in antibiotic use in regions where effective immunoprophylaxis is available (Rathor and Swain, 2024). Conventional vaccination strategies, including immersion and intraperitoneal injection, induce robust systemic immune responses but require individual handling, limiting their application to early life stages or single-dose regimens (Bøgwald and Dalmo, 2021).

To overcome these constraints, mucosal vaccination, particularly oral delivery, has gained increasing attention. Oral vaccines enable mass immunization with minimal handling stress and represent the only practical approach for revaccinating adult fish reared in sea cages during prolonged production cycles (Muñoz-Atienza, 2021). In parallel, vaccine platforms have diversified to include DNA vaccines, novel adjuvants, and nanocarrier-based systems aimed at enhancing innate immune activation, antigen uptake by antigen-presenting cells, and the durability of protective responses (Mutoloki et al., 2015). Despite these technological advances, vaccine performance in teleost aquaculture remains variable under field conditions, and disease-associated losses persist even in vaccinated populations. A key limitation is that many fish vaccines have historically been developed using mammalian immunological paradigms, with insufficient consideration of teleost-specific immune organization and regulation (Yu et al., 2020). Although teleost fish possess both innate and adaptive immune components, fundamental differences in antibody repertoires, pattern-recognition receptor signaling, and the relative contribution of innate immunity critically influence the development of protective responses (Campbell et al., 2023).

These conceptual and biological limitations are particularly evident in Atlantic salmon affected by SRS. In this context, vaccination has proven insufficient to confer consistent protection under field conditions, highlighting a persistent mismatch between current vaccine designs and the immunological requirements for controlling chronic, intracellular bacterial infections in teleost fish such as *P. salmonis*.

## 3 Antibiotic dependence in Chilean salmon farming

Chile is the second-largest salmon producer worldwide, and its production system operates through a biphasic model. Fish are initially reared in freshwater hatcheries until smoltification and subsequently transferred to seawater cages, where high stocking densities and fluctuating environmental conditions increase pathogen transmission. This production structure has strongly shaped patterns of antibiotic use in Chilean salmon farming over the past two decades. Unlike other major producing regions, Chilean salmon aquaculture has faced a distinct epidemiological landscape characterized by recurrent bacterial outbreaks. Early challenges included furunculosis caused by *Aeromonas salmonicida* (Godoy et al., 2010) and bacterial cold-water disease associated with *Flavobacterium psychrophilum* (Avendaño-Herrera et al., 2014). However, the most persistent and consequential threat has been *P. salmonis*, first reported in Chile in 1989 (Bravo and Campos, 1989; Cvitanich et al., 1991). SRS is now responsible for most bacterial mortalities during the seawater grow-out phase, and the limited efficacy of available vaccines against this intracellular pathogen has driven the widespread adoption of metaphylactic antibiotic treatments, primarily florfenicol and oxytetracycline (Rozas-Serri, 2022).

A critical turning point in the evolution of Chilean salmon farming was the infectious salmon anemia (ISA) crisis between 2007 and 2010 (Godoy et al., 2008). Although ISA is a viral disease and did not require antibiotic treatment, the outbreak exposed major structural weaknesses in the production model, including excessive farm density, inadequate coordination among sites, insufficient fallowing, and unrestricted movement of fish and personnel (Asche et al., 2009). The resulting production instability increased susceptibility to secondary bacterial infections and intensified antimicrobial use as a risk-mitigation strategy (Henriksson et al., 2017). In response, Chile implemented a comprehensive reform of its sanitary governance framework. Among the most significant measures was the establishment of the *Agrupaciones de Concesiones Salmoneras* (Salmon Concession Groups, ACS), designed to coordinate synchronized production cycles and fallowing among neighboring farms to reduce pathogen persistence and transmission. Central to this new architecture was the *Programa de Gestión Sanitaria de la Acuicultura* (Aquaculture Health Management Program, PGSA), conceived as an integrated framework for the surveillance, prevention, and control of infectious diseases throughout the production cycle, adopting a systemic approach to aquatic animal health. The PGSA integrates four complementary areas of action. From an epidemiological perspective, it mandates compulsory surveillance and risk-based monitoring of morbidity and mortality. In microbiology and parasitology, it emphasizes laboratory confirmation of pathogens to support evidence-based sanitary decisions. Host-related factors, including species, life stage, and physiological or environmental stress, are incorporated to contextualize disease susceptibility. Finally, from a pharmacological standpoint, the PGSA links antimicrobial use to confirmed diagnoses, veterinary oversight, and regulatory compliance, promoting more rational and traceable antibiotic use.

Building on this framework, the *Programa Sanitario Específico de Vigilancia y Control de Piscirickettsiosis* (Specific Health Program for Surveillance and Control of Piscirickettsiosis, PSEVC) established systematic monitoring and risk-based classification for SRS, the primary driver of antibiotic consumption in Chilean salmon farming. In parallel, the *Sistema de Información para la Fiscalización de la Acuicultura* (Aquaculture Enforcement Information System, SIFA) centralized the reporting of health events, production metrics, and antimicrobial prescriptions, substantially improving traceability and transparency. Together, these instruments marked a transition from a predominantly reactive disease-control model toward a more structured, regulated, and data-driven system.

Despite these regulatory advances and the expansion of preventive strategies, antibiotic therapy has remained the main tool for controlling bacterial diseases in Chilean salmon aquaculture. Antimicrobial use continues to be driven by a combination of environmental pressures, including high stocking densities, temperature variability, low oxygen levels, and episodic harmful algal blooms, and pathogen prevalence (Upmanyu and Malviya, 2020; Arriagada et al., 2024; Carrasco-Bahamonde and Casellas, 2024).

Historically, antibiotic use in Chilean salmon farming has evolved in response to successive disease pressures. During the early expansion of the industry, multiple antimicrobial classes, including quinolones, were widely used. However, increasing concerns regarding antimicrobial resistance, food safety, and international trade led to the prohibition of quinolones in 2012, marking a major shift in antimicrobial policy (SERNAPESCA, 2012; Collignon et al., 2016; Schmerold et al., 2023). Following this ban, therapeutic options became progressively restricted, consolidating reliance on a reduced number of compounds. Within this constrained therapeutic landscape, florfenicol emerged as the dominant antimicrobial due to its broad-spectrum activity and favorable pharmacokinetic properties in salmonids, while oxytetracycline remained a complementary option, particularly during freshwater stages (San Martín et al., 2019; Trincado et al., 2024). Over time, florfenicol became the principal antibiotic used during seawater production, where SRS pressure is highest, whereas antibiotic use in freshwater hatcheries remained comparatively lower and primarily targeted acute juvenile infections. A notable exception occurred between 2013 and 2015, when outbreaks of *F. psychrophilum* led to a transient increase in freshwater antibiotic use.

Overall, this historical trajectory demonstrates that Chile’s current dependence on antibiotics is not the result of isolated management decisions, but rather the cumulative outcome of long-term interactions among production intensity, environmental stressors, pathogen ecology, regulatory transformations, and limited preventive alternatives. Within this framework, the sustained dominance of florfenicol and oxytetracycline must be understood as a systemic response to unresolved structural and biological challenges in Chilean salmon aquaculture.

## 4 Antibiotic treatment strategies and routes of administration

**Figure 2:**
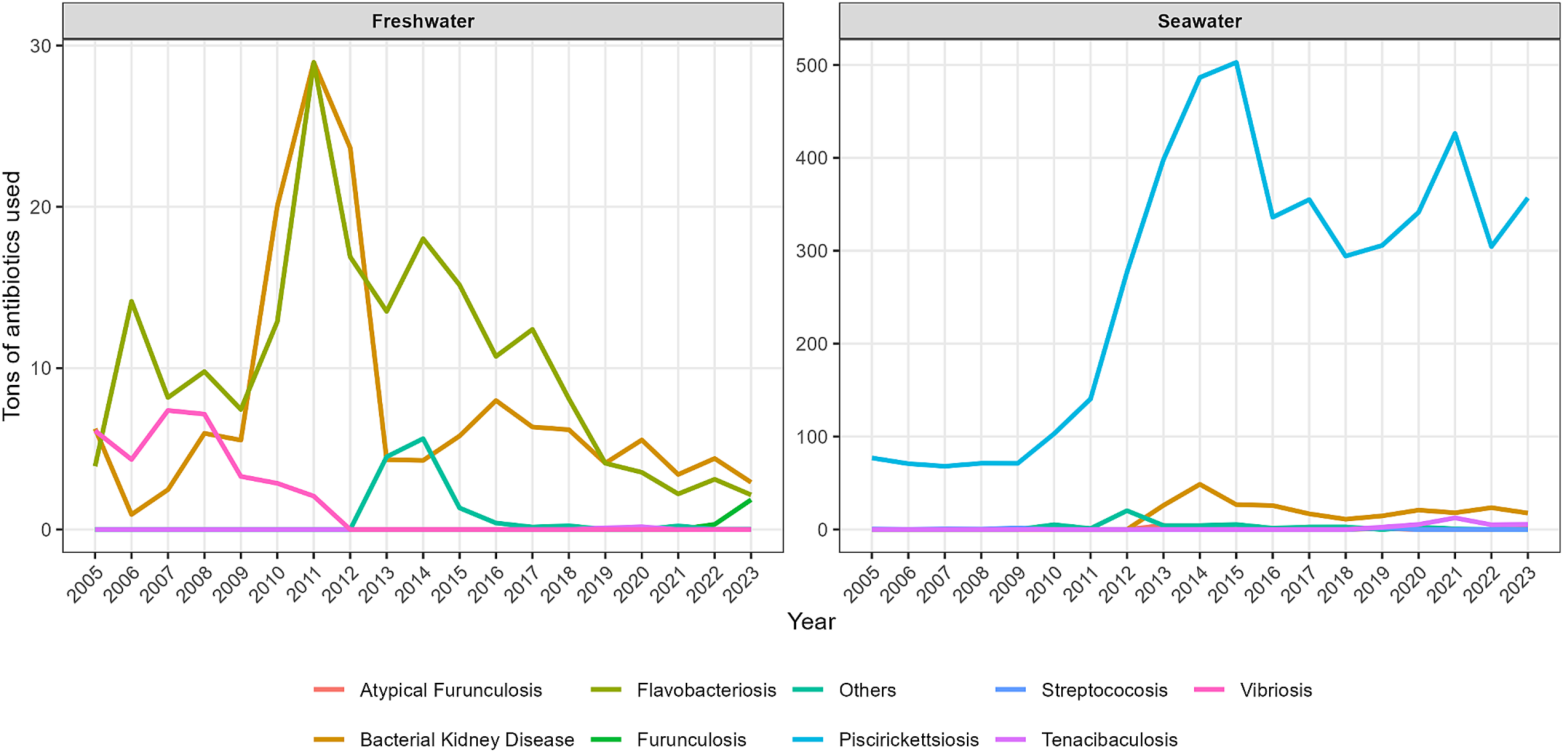
Temporal trends in antimicrobial use in Chilean salmon aquaculture by production phase (2006–2023). Line plots show annual antimicrobial use, expressed as kilograms of active ingredient, in the freshwater and seawater production phases, disaggregated by antibiotic class. A marked transient peak is evident in the freshwater phase between 2013 and 2015, corresponding to outbreaks of *Flavobacterium psychrophilum* in hatchery systems. In the seawater phase, antimicrobial use remained substantially higher and was dominated primarily by florfenicol and oxytetracycline, underscoring the persistent burden of *Piscirickettsia salmonis* as a major health challenge during marine grow-out.

Antibiotic treatment strategies in salmon aquaculture are shaped by the production cycle, disease pressure, and operational feasibility. Salmon production is broadly divided into two main stages, the freshwater phase and the seawater grow-out phase, each with distinct patterns of antimicrobial use (Henriksson et al., 2015; Cabello and Godfrey, 2023) (Figure 2). During the freshwater phase, fish are reared from early fry to smolts under relatively controlled conditions. At this stage, antibiotic use is mainly associated with the treatment of acute bacterial infections, including those caused by *F. psychrophilum* and *Yersinia ruckeri* (Troncoso et al., 2010; Miranda et al., 2016). The lower total volume of antibiotics used in freshwater, compared with the seawater phase, largely reflects the smaller body weight and lower total biomass of fish rather than a lower therapeutic need (SERNAPESCA, 2025). Treatments are administered primarily through medicated feed and, to a lesser extent, by immersion baths, which remain operationally feasible because of the smaller fish size and more controlled water volumes (Rairat et al., 2023; Au-Yeung et al., 2025). Historically, oxytetracycline has been the most widely used antibiotic during this phase (SERNAPESCA, 2025).

By contrast, the seawater grow-out phase accounts for most antibiotic consumption in Chilean salmon farming. High stocking densities, environmental variability, and prolonged exposure to pathogens, particularly *P. salmonis*, generate sustained disease pressure during this stage (Rozas and Enríquez, 2014; Hillman et al., 2020). As a result, florfenicol has become the dominant antimicrobial, administered almost exclusively through medicated feed to large populations reared in marine cages (Miranda et al., 2018; SERNAPESCA, 2025).

From a conceptual perspective, antibiotic use in aquaculture can be categorized as prophylactic, metaphylactic, or targeted (therapeutic). Prophylactic treatment involves antibiotic administration before the onset of clinical disease. Metaphylaxis refers to treatment of an entire population after disease has been detected in a subset of individuals, with the aim of reducing mortality and limiting pathogen spread (Sørum, 2008; Love et al., 2020). Targeted therapy, in contrast, is directed toward clinically affected individuals or defined groups following diagnostic confirmation. In practice, these strategies are closely linked to the route of administration. Prophylactic and metaphylactic treatments are usually delivered through medicated feed or immersion baths, whereas targeted therapy may involve injection, particularly in broodstock or other high-value fish (Rostang et al., 2021). However, individual treatment is rarely feasible at commercial scale because of logistical constraints and animal welfare considerations (Sørum, 2008). In Chile, prophylactic antibiotic use has been prohibited since 2016, and antimicrobials may be administered only for therapeutic or metaphylactic purposes under veterinary prescription and laboratory-confirmed diagnosis (SERNAPESCA, 2015; OIE, 2020). In the case of SRS, metaphylactic treatment is authorized when a salmon farm or a specific cage reaches or exceeds a weekly mortality threshold of 0.35% attributable to *P. salmonis*, as confirmed by diagnostic testing (SERNAPESCA, 2012, 2021). This threshold is used as an operational epidemiological criterion to support timely intervention and limit disease progression (Hillman et al., 2020). Nevertheless, the 0.35% mortality threshold should be interpreted primarily as a regulatory and operational trigger rather than as a biologically validated indicator of treatment necessity or expected efficacy. To date, no publicly available evidence has demonstrated that this threshold has predictive validity in terms of improving survival outcomes or reducing cumulative antimicrobial use. This limitation is important because treatment decisions based exclusively on mortality may fail to reflect the underlying infection dynamics or the stage of disease progression within the population. These shortcomings highlight the need to incorporate epidemiological, pharmacokinetic, and biological parameters into antimicrobial decision-making frameworks.

A major limitation of metaphylaxis during the seawater phase lies in its predominant administration through medicated feed. Under this approach, antibiotic exposure depends directly on feed intake. Because anorexia or reduced appetite is a common manifestation of disease, clinically affected fish are likely to consume lower amounts of medicated feed (Rozas and Enríquez, 2014). In contrast, healthy or subclinically infected fish, that is, individuals carrying the pathogen without overt clinical signs or behavioral changes, usually maintain normal feeding behavior and therefore receive proportionally higher antibiotic doses. This creates a heterogeneous distribution of antimicrobial exposure across the population, reducing therapeutic efficacy in diseased fish while unnecessarily exposing non-diseased individuals. Such conditions favor subtherapeutic exposure and increase the selective pressure for antibiotic-resistant bacteria (Andersson and Hughes, 2014; Price et al., 2019). In addition, the total dose and duration of metaphylactic treatment are determined by the attending veterinarian, without legally binding limits on the maximum dose per treatment, the number of treatments per production cycle, or the cumulative exposure of non-diseased fish (SERNAPESCA, 2015; San Martín et al., 2021). This regulatory framework increases the risk of repeated and suboptimal exposure at both individual and population levels.

Recent experimental evidence further indicates that repeated exposure to florfenicol through medicated feed can induce intestinal dysbiosis, alter inflammatory responses, and negatively affect growth parameters in healthy Atlantic salmon, even in the absence of clinical infection (Camacho-Méndez et al., 2025). These findings suggest that metaphylaxis, as currently implemented, may generate unintended biological effects on the host, disrupt intestinal homeostasis, and contribute to recurrent intervention cycles. At the same time, a substantial fraction of orally administered antibiotics is released into the environment through uneaten feed and fecal excretion, leading to accumulation in sediments and dispersion into the surrounding water column (Buschmann et al., 2012; Shah et al., 2014; Ortiz-Severín et al., 2025). These emissions impose selective pressure on environmental microbial communities, promoting the emergence and persistence of antibiotic-resistant bacteria and resistance genes, with broader ecological and public health implications within a One Health framework (Olivares et al., 2013; Kraemer et al., 2019).

Taken together, although metaphylaxis remains a legally permitted and operationally necessary strategy in Chilean salmon farming, its reliance on mortality-based triggers, its predominantly oral route of administration, and the absence of explicit regulatory criteria for dosage and treatment frequency underscore the need for more restrictive authorization schemes and more precise, preventive disease-control strategies.

## 5 Impact of antibiotics on aquatic systems

Once released into aquatic environments, antibiotics exert selective pressure on microbial communities, promoting the emergence and persistence of antibiotic-resistant bacteria (O’Malley et al., 2024). These effects extend beyond target pathogens and affect non-target organisms, including invertebrates and microorganisms that play essential roles in ecosystem functioning (Tang et al., 2024). Changes in microbial community composition can disrupt key biogeochemical processes, such as nitrogen cycling and organic matter decomposition, thereby impairing primary productivity and overall ecosystem stability (Tello et al., 2010).

In Chilean salmon farming zones, these concerns are particularly relevant because antibiotics are administered mainly through medicated feed, and a substantial fraction may reach surrounding marine compartments through fecal excretion and uneaten pellets. Oxytetracycline is of special concern due to its persistence in sediments. Experimental and environmental evidence discussed in Chilean studies indicates that oxytetracycline can remain in sedimentary matrices for prolonged periods, with reported half-lives exceeding 100 days and even longer persistence in interstitial waters and deeper sediment layers, supporting the view that benthic environments can function as long-term reservoirs of antimicrobial exposure (Ahumada-Rudolph et al., 2016, 2021). In southern Chile, this persistence has motivated interest in bioremediation strategies, including the use of marine fungi isolated from fjord sediments beneath salmon farming influence, several of which showed substantial oxytetracycline degradation capacity under laboratory conditions (Ahumada-Rudolph et al., 2016, 2021).

Although Chile-specific sediment-effect thresholds remain poorly resolved, available evidence indicates that ecological risk cannot be interpreted solely from antibiotic presence or absence. Organic enrichment from aquaculture wastes interacts with chemotherapeutic inputs and may strongly modulate benthic microbial responses. Experimental sediment microcosm studies in aquaculture settings have shown that organic matter enrichment can drive marked shifts in bacterial community structure, while oxytetracycline at environmentally relevant concentrations may not always produce readily detectable taxonomic changes over short observational windows, suggesting that community-level effects may depend on concentration, sediment properties, exposure time, and endpoints evaluated (Johnson et al., 2023). These findings are important when interpreting Chilean conditions, where high organic loading beneath cages may act together with antimicrobial residues to shape benthic ecosystem responses.

Evidence from southern Chile further shows that the ecological footprint of antibiotic use extends beyond production sites. In coastal wetlands of Chiloé, 62% of sediment samples contained antibiotic-resistant bacteria, and multi-resistant phenotypes were detected only in sediments from the bay influenced by aquaculture operations. The same study also reported shared resistance markers between sediments and migratory shorebirds, including genes such as *floR* and *bla_TEM_*, supporting the idea that salmon aquaculture can contribute to a broader environmental resistome that reaches wildlife and protected coastal systems (Navedo et al., 2021). Notably, the authors emphasized that these findings were likely conservative, since sediments closer to salmon aquaculture operations would be expected to contain higher antibiotic residues and a stronger antibiotic footprint (Navedo et al., 2021).

Antibiotics can also persist and accumulate in aquatic sediments, prolonging the exposure of benthic organisms to sub-lethal concentrations. Chronic exposure has been associated with physiological stress, altered behavior, and reduced fitness in non-target species, with potential consequences for population dynamics and predator–prey interactions (Brodin et al., 2014; Kovalakova et al., 2020; Yang et al., 2022). For example, antibiotic-induced behavioral changes in zooplankton, key components of aquatic food webs, may propagate across trophic levels and ultimately affect fish populations and broader ecosystem stability (Yamamuro et al., 2019; Yang et al., 2020). Collectively, these disturbances may compromise ecosystem services such as water purification, nutrient regulation, and habitat provision, which are fundamental to both ecological integrity and human well-being (Grizzetti et al., 2019).

In addition to ecological effects, the bioaccumulation of antibiotics in aquatic organisms poses risks to higher trophic levels, including humans (Kovalakova et al., 2020; Zhu et al., 2024). Through the consumption of fish and other aquatic products, antibiotics and their metabolites may move across the food web, raising concerns related to resistance selection and dissemination, as well as potential adverse health effects such as allergic reactions (Liu et al., 2017; Manage, 2018). Under a One Health framework, the Chilean case is especially relevant because sediment-associated resistance may not remain confined to farming sites but may spread across coastal environments and into wildlife-associated microbiota (Navedo et al., 2021).

An additional gap in the Chilean context is the limited availability of site-specific ecological benchmarks, such as sediment quality thresholds or PNEC-based (Predicted No-Effect Concentration) interpretations, for the antibiotics most heavily used in salmon aquaculture. Likewise, although fallowing is widely used as a mitigation measure, available Chilean evidence remains insufficient to define recovery trajectories for antibiotic residues, benthic microbial communities, and resistance markers after production cycles. This lack of integrated ecological baselines limits the ability to determine when sediment recovery is truly achieved and highlights the need for longitudinal monitoring that combines residue measurements, microbial community analyses, and ARG surveillance under Chilean field conditions.

In summary, the release of antibiotics from aquaculture into aquatic environments produces wide-ranging ecological effects, altering microbial communities, affecting non-target organisms, and potentially compromising biodiversity and ecosystem functioning. In Chile, these concerns are reinforced by evidence of oxytetracycline persistence in sediments, the occurrence of resistant bacteria and resistance genes in coastal systems influenced by salmon farming, and the potential for environmental dissemination beyond farm boundaries. These findings underscore the need for more sustainable disease-control strategies, including improved vaccination, better husbandry, strengthened regulation, systematic environmental surveillance, and the development of remediation or recovery-oriented approaches for impacted sediments.

## 6 AMR phenomenon in the Chilean salmon industry

Antimicrobial resistance (AMR) in the Chilean salmon industry is a multifaceted challenge with implications across three interconnected dimensions: (i) resistance in primary fish pathogens, which may compromise therapeutic efficacy; (ii) resistance in opportunistic fish and human pathogens, with potential public health consequences; and (iii) resistance within microbiota associated with farming systems, which serve as reservoirs and vectors of antibiotic resistance genes (ARGs). Each dimension poses distinct epidemiological and management challenges, as outlined below.

### 6.1 Resistance in fish pathogens

*Piscirickettsia salmonis* exemplifies a particularly relevant paradox in Chilean salmon aquaculture. Despite sustained and intensive antibiotic use, current evidence does not support the widespread acquisition of horizontally transferred antimicrobial resistance genes as the main explanation for recurrent therapeutic limitations. To date, the multidrug resistance plasmid p3PS10 remains the clearest example of acquired resistance in this pathogen, demonstrating that horizontal gene acquisition is possible, although apparently uncommon (Pulgar et al., 2015; Bohle et al., 2017; Saavedra et al., 2018). This interpretation is further supported by comparative genomic analyses of 73 high-quality genomes from Chilean and international isolates, which found no evidence that treatment failure in piscirickettsiosis is broadly explained by horizontal acquisition of resistance genes (Schober et al., 2023).

This apparent paradox indicates that reduced treatment efficacy in *P. salmonis* should be interpreted within a broader mechanistic framework that distinguishes among acquired genetic resistance, adaptive tolerance, and treatment failure. First, true acquired resistance may occur in specific isolates, as illustrated by p3PS10 (Saavedra et al., 2018). Second, tolerance-like phenotypes may emerge without stable acquisition of classical ARGs. In this context, nutrient limitation has been shown to induce medium-dependent metabolic resistance to multiple antibiotics (Schober et al., 2023; Escalona et al., 2026), indicating that physiological state can strongly influence susceptibility. In addition, recent evidence shows that sub-inhibitory concentrations of florfenicol modulate the expression of biofilm-associated and resistance-associated genes in biofilm-embedded *P. salmonis*, including the efflux pump *acrAB* and the two-component systems *cpxAR* and *qseBC*, supporting the idea that suboptimal antibiotic exposure may promote adaptive responses linked to persistence and reduced susceptibility (Escalona et al., 2026).

Third, treatment failure may also result from pharmacodynamic and operational constraints rather than from resistance sensu stricto. *P. salmonis* is a facultative intracellular pathogen, a trait that complicates effective antimicrobial delivery at the site of infection and may also help explain the limited protection conferred by current commercial vaccines under field-like challenge conditions (Figueroa et al., 2022; Vargas-Chacoff et al., 2025). In parallel, oral metaphylaxis can generate heterogeneous antibiotic exposure at the population level, favoring sub-therapeutic concentrations in diseased fish with reduced feed intake while unnecessarily exposing non-clinical individuals (Baptiste and Kyvsgaard, 2017; Sáenz et al., 2019). Under such conditions, poor clinical response may reflect inadequate drug exposure, intracellular protection, biofilm-associated tolerance, or persistence-related phenotypes rather than stable acquired resistance alone (Ortiz-Severín et al., 2021; Farias et al., 2025). Altogether, these findings indicate that the AMR problem *in P. salmonis* should not be framed exclusively in terms of classical ARG acquisition, but rather as the result of interacting genetic, physiological, ecological, and therapeutic factors.

Other fish pathogens relevant to Chilean salmon farming have also shown reduced antimicrobial susceptibility. *Y. ruckeri* has been identified as the sole etiological agent in outbreaks affecting Chilean salmonids, with isolates resistant to penicillin and erythromycin, suggesting clonal dissemination (Troncoso et al., 2010). Similarly, *F. psychrophilum* isolates from Chile exhibited decreased susceptibility to oxytetracycline and quinolones based on MIC testing, which proved more reliable than disk diffusion and revealed an increased risk of treatment failure (Miranda et al., 2016). MLST analyses demonstrated that Chilean *F. psychrophilum* genotypes are closely related to European and North American lineages, with large-scale egg importation and mixed-species farming likely facilitating strain introduction and dissemination (Avendaño-Herrera et al., 2014). More recently, the expansion of antigenic groups from four to fourteen using a PCR-based serotyping approach has further highlighted pathogen diversity in Chile and the need to adapt management practices accordingly (Avendaño-Herrera et al., 2020).

Additional bacterial pathogens also warrant consideration. *Tenacibaculum* spp., associated with tenacibaculosis in marine-reared salmonids, are increasingly recognized as contributors to ulcerative and necrotic skin diseases during the seawater phase. Although systematic AMR surveillance for *Tenacibaculum* spp. remains limited in Chile, reports of reduced susceptibility to commonly used antimicrobials raise concerns regarding underrecognized treatment failure and continued selection pressure (Avendaño-Herrera et al., 2022). Likewise, *Renibacterium salmoninarum* (BKD) remains problematic due to its chronic intracellular lifestyle and vertical transmission, which complicate detection and have historically driven prolonged or repeated antimicrobial exposure with limited efficacy (Delghandi et al., 2020).

### 6.2 Opportunistic human pathogens and public health linkages

Beyond fish pathogens, salmon-farming environments can support opportunistic bacteria with zoonotic potential and clinically relevant resistance determinants, establishing a plausible interface between aquaculture, coastal microbiomes, and human health. This issue is particularly relevant in regions of intensive salmon aquaculture in southern Chile, where marine and freshwater compartments are repeatedly exposed to antimicrobials and high-density animal production, creating conditions that may enrich and maintain resistance genes in environmental bacteria (Cabello, 2006; Buschmann et al., 2012; Shah et al., 2014; Lozano-Muñoz et al., 2021).

Evidence supporting a connection between aquaculture-associated resistomes and human pathogens has been reported for plasmid-mediated quinolone resistance determinants. In a study comparing quinolone-resistant *Escherichia coli* from urinary tract infections, isolates from patients in Chile’s aquaculture region were significantly enriched for *qnr* genes, including *qnrB*, *qnrS*, and *qnrA*, as well as *aac(6′)-Ib*, relative to isolates from a non-aquaculture comparator region. Importantly, *qnrA1*, *qnrB1*, and *qnrS1* sequences detected in quinolone-resistant Chilean *E. coli* were identical to those found in Chilean marine bacteria, consistent with horizontal gene transfer between aquatic environmental bacteria and human pathogens (Tomova et al., 2015). Complementing these findings, studies on quinolone-resistance determinants in Chile have shown that aquaculture-associated marine bacteria can carry *qnr*-related genes capable of conferring decreased quinolone susceptibility when expressed in *E. coli*. For example, a *qnrA*-related determinant from clinical *Vibrio parahaemolyticus* was shown to reduce quinolone susceptibility after cloning into *E. coli* K-12, supporting the biological plausibility that resistance determinants circulating in marine bacterial communities may be functionally relevant in enteric bacteria (Aedo et al., 2014).

Taken together, these findings provide compelling evidence of shared resistance determinants across environmental and clinical compartments. From a One Health perspective, however, they do not imply direct attribution of clinical AMR to salmon farming alone. Rather, they support the view that aquaculture-impacted coastal ecosystems can function as reservoirs and exchange networks for mobile resistance determinants that are also encountered in human pathogens. This underscores the need for integrated surveillance strategies that jointly consider (i) farm-associated environmental bacteria, (ii) opportunistic bacteria and indicator organisms such as *E. coli*, and (iii) clinically relevant resistance markers, including *qnr* and other mobile elements, particularly in high-production coastal regions where environmental exposure, antimicrobial use, and human interaction converge to create conditions favorable for resistance dissemination.

Conceptual models have been proposed to describe potential pathways linking antimicrobial use in aquaculture to human-associated antimicrobial resistance through environmental dissemination, food contamination, and clinical exposure (Salgado-Caxito et al., 2022). These frameworks illustrate connectivity between aquatic environments and human health; however, they remain largely hypothetical rather than demonstrating confirmed transmission pathways.

**Figure 3:**
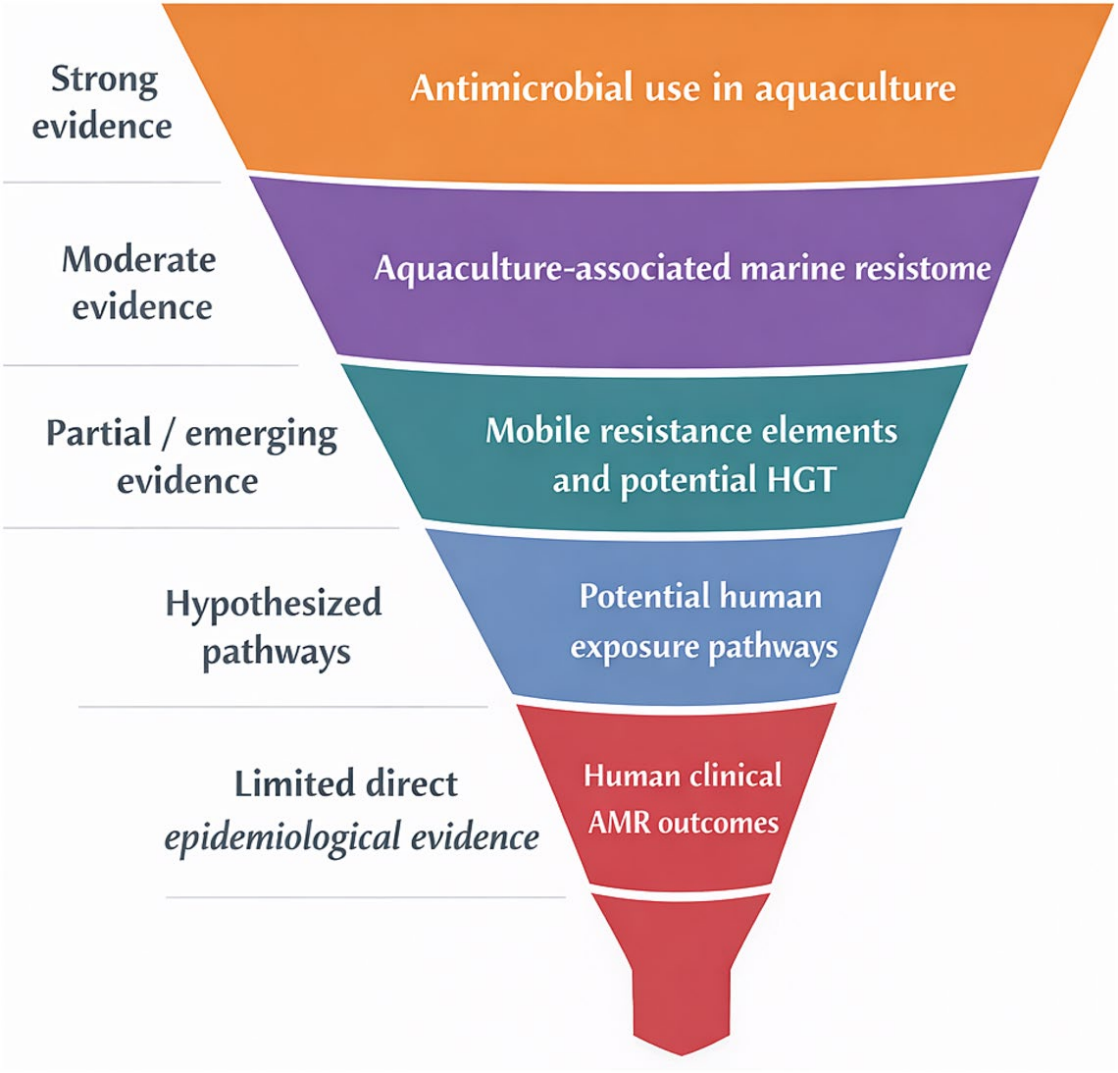
Conceptual evidence funnel linking antimicrobial use in aquaculture to human clinical antimicrobial resistance (AMR). The funnel illustrates a progressive pathway from antimicrobial use in aquaculture to aquaculture-associated marine resistomes, mobile resistance elements, and potential horizontal gene transfer, possible human exposure pathways, and human clinical AMR outcomes. Funnel narrowing represents the progressive decline in evidentiary strength across the pathway: evidence is strongest for antimicrobial selection and resistance enrichment in aquaculture environments, moderate for shared resistance determinants in marine bacteria, and increasingly limited for direct attribution to human exposure and clinical outcomes. The model emphasizes that ecological connectivity and genetic overlap support biological plausibility, but do not by themselves demonstrate confirmed epidemiological transmission.

Importantly, the presence of shared resistance determinants, such as plasmid-mediated *qnr* genes, in marine bacteria and clinical *E. coli* suggests potential genetic exchange across ecological compartments but does not constitute direct evidence of transmission. Attribution of antimicrobial resistance across environmental and human systems remains limited by methodological constraints, including the difficulty of distinguishing horizontal gene transfer from independent selection processes under similar antimicrobial pressures (Figure 3).

Overall, current evidence supports the existence of interconnected resistomes across marine and human-associated environments, but epidemiological evidence linking aquaculture-derived resistance to clinical outcomes remains limited.

### 6.3 Resistance in the microbiota of farming systems

The salmon farming environment, including gut microbiota, sediments, and the surrounding water column, constitutes a major reservoir of ARB and ARGs. *Pseudomonas* spp. isolated from Atlantic salmon (*Salmo salar*) under antibiotic treatment have displayed extremely high MICs for florfenicol (≥2048 μg/mL) and oxytetracycline (≥1,024 μg/mL), with most isolates carrying corresponding ARGs and class 1 integrons, highlighting their role as reservoirs and disseminators of AMR (Higuera-Llantén et al., 2018; Vásquez-Ponce et al., 2022).

Since 2002, studies have reported oxytetracycline-resistant and multidrug-resistant bacteria in Chilean salmon farms, including isolates resistant to up to ten antimicrobials, among them compounds critically important for human medicine such as ampicillin and erythromycin (Miranda and Zemelman, 2002). More recent surveys of Chilean marine bacteria found that 81% of isolates from aquaculture and control sites were resistant to at least one antibiotic, with *tetA* and *tetG* identified as key drivers of tetracycline resistance, suggesting that selective pressure extends even to areas distant from farming sites (Shah et al., 2014). Metagenomic profiling identified *floR* as the predominant phenicol-resistance gene, with sequence diversity indicating the coexistence of high- and low-resistance strains in the environment (Ortiz-Severín et al., 2025). Most Chilean studies have relied on culture-based and PCR-based detection of ARGs, which effectively capture known targets but may overlook novel genes and unculturable bacteria. Although still underutilized in Chile, metagenomics offers a broader view of resistome diversity, although it cannot always link genes to hosts or confirm gene expression (Lanza et al., 2018).

Sediment studies in Chilean seawater farms have revealed multidrug-resistant isolates carrying plasmids with quinolone (*qnrA*, *qnrB*, *qnrS*) and tetracycline (*tetA*, *tetB*, *tetK*) resistance genes (Buschmann et al., 2012), as well as class 1 integrons and TEM-type *β*-lactamases (Shah et al., 2014). Importantly, conjugation assays demonstrated that plasmids from salmon farm environments could transfer quinolone resistance determinants to uropathogenic *E. coli*. Subsequent studies confirmed mobile genetic elements of aquaculture origin in human pathogens, with the potential to compromise ciprofloxacin therapy (Aedo et al., 2014; Tomova et al., 2015, 2018).

Together, these findings indicate that the resistome associated with Chilean salmon farming represents not only an environmental concern but also a potential public health risk, reinforcing the urgent need for systematic monitoring and stricter antibiotic stewardship.

## 7 Regulation and policies

The Chilean salmon industry operates under a comprehensive and continuously evolving legal framework designed to promote sustainable production, safeguard animal health, and protect public health (Law 18892, 1989 and subsequent amendments). The National Fisheries and Aquaculture Service (in Spanish: *Servicio Nacional de Pesca y Acuicultura*, SERNAPESCA) serves as the main regulatory authority, overseeing enforcement, inspections, and compliance with national and international sanitary standards (SERNAPESCA, 2025).

Antimicrobial use in Chilean salmon aquaculture is regulated through the General Health Program for Antimicrobial Use in Salmoniculture and Other Cultured Fish, established as part of the broader regulatory reform implemented after the infectious salmon anemia (ISA) crisis (2007–2010). This program integrates epidemiological surveillance, microbiological and diagnostic confirmation, host-related risk factors, and pharmacological oversight into a unified regulatory framework. Its main objective is to mitigate antimicrobial resistance risks while maintaining fish health and production efficiency through prudent, justified, and traceable antimicrobial use (SERNAPESCA, 2015, 2025). A central component of this framework is the requirement that antimicrobial treatments be prescribed exclusively under veterinary supervision and supported by clinical and diagnostic justification. Metaphylactic treatments are permitted only under scientifically justified conditions and must comply with internationally recognized guidelines (SERNAPESCA, 2015; OIE, 2020). Veterinarians and accredited professionals registered with the competent authorities are required to maintain detailed treatment records, including indication, dosage, duration, treated biomass, and therapeutic outcomes. These records are subject to routine audits and inspections conducted by SERNAPESCA (2025). Regulations further stipulate that antimicrobials be administered at the lowest effective dose and for the shortest duration necessary, with diagnostic support, such as antimicrobial susceptibility testing, recommended to ensure appropriate drug-pathogen matching (San Martín et al., 2021; SERNAPESCA, 2021).

The ISA virus crisis between 2007 and 2010 exposed major structural weaknesses in Chilean salmon aquaculture, including insufficient biosecurity measures, limited epidemiological surveillance, and poor coordination among farming sites, ultimately triggering a comprehensive restructuring of the regulatory system (Godoy et al., 2008). In response, Chile introduced the Salmon Concession Groups (ACS) in 2010, mandating synchronized production cycles, coordinated sanitary management, and shared epidemiological responsibility among neighboring farms within defined geographic areas (Ibieta et al., 2011). Building on this area-based approach, the PGSA was implemented as a national framework to standardize surveillance, diagnostic confirmation, and sanitary reporting across the industry. Subsequently, the Specific Health Program for Surveillance and Control of Piscirickettsiosis (PSEVC) was approved in 2012 following the classification of piscirickettsiosis as a high-risk disease under Supreme Decree No. 319 (Ministerio de Economía y SUBPESCA, 2001). The PSEVC established mandatory biweekly PCR surveillance for *P. salmonis*, risk-based farm classification, veterinary audits, and laboratory confirmation as prerequisites for antimicrobial treatment authorization (SERNAPESCA, 2012).

Antimicrobial use in Chilean salmon aquaculture must be reported through the Aquaculture Enforcement Information System (SIFA), a compulsory digital platform that records prescriptions, antimicrobial class, dosage, duration, treated biomass, and production cycle. Importantly, antimicrobial use data are aggregated and reported annually at the ACS level, forming the basis of SERNAPESCA’s official Annual Antimicrobial Use Report. This reporting system provides transparency, enables interannual comparisons, and supports regulatory oversight at both regional and national scales (SERNAPESCA, 2025).

This area-based reporting strategy aligns Chile with other major salmon-producing countries. Norway, for example, reports annual antimicrobial use at the national level through the Norwegian Veterinary Institute, while Canada and Scotland similarly publish sector-wide antimicrobial use statistics integrated into regulatory surveillance systems. However, Chile remains distinctive in the scale and intensity of antimicrobial use reported, underscoring the need for continued regulatory refinement, strengthened preventive strategies, and systematic international benchmarking to reduce antimicrobial dependence while maintaining production viability.

## 8 Quantitative assessment of antimicrobial use trends

To further contextualize the international differences in antibiotic use described above, we conducted a temporal trend analysis using antimicrobial consumption normalized as kilograms of active ingredient per ton of biomass produced. This approach enables direct comparison among salmon-producing countries with different production scales and management strategies.

Clear differences in temporal patterns were observed across countries. In Chile, antibiotic use showed a moderate downward trend over time (r = −0.64); however, this association did not reach statistical significance (*p* = 0.065), indicating that the observed reductions have not followed a consistent trajectory. Instead, antimicrobial use displayed marked interannual variability. By contrast, Norway and Canada exhibited stable patterns over time, with no significant trends detected (r = 0.02, *p* = 0.96; r ≈ 0.00, *p* ≈ 0.999, respectively), reflecting persistently low levels of antimicrobial use.

**Figure 4:**
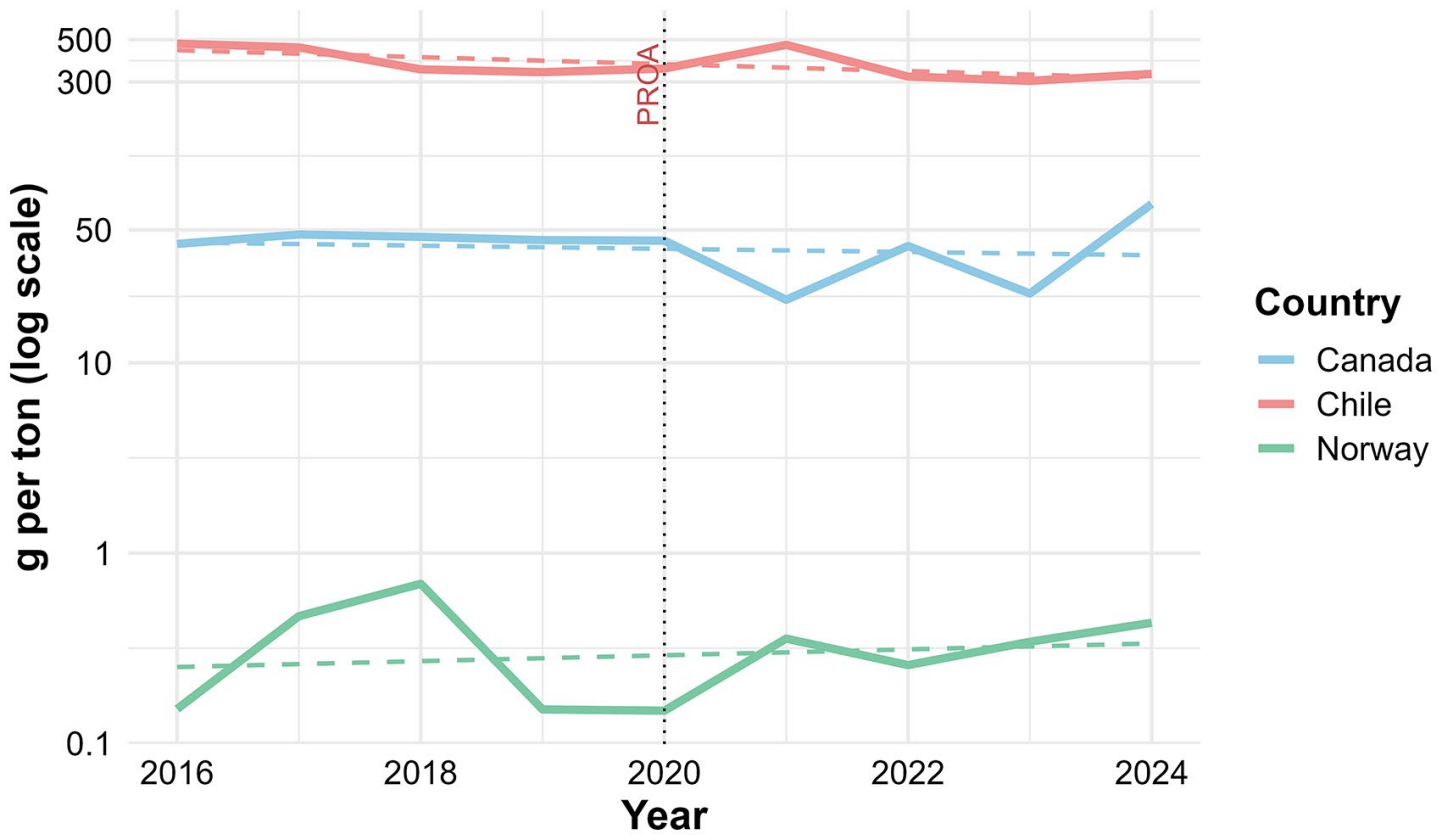
Temporal trends in antimicrobial use across major salmon-producing countries. Temporal trends in antimicrobial use (AMU), expressed as grams of active ingredient per ton of biomass, in Chile, Norway, and Canada over the study period. Solid lines represent annual AMU values, whereas dashed lines indicate the linear regression trend for each country. Chile shows an overall decreasing tendency over time, but with marked interannual variability, while Norway and Canada maintain consistently low and stable AMU levels. The vertical dotted line indicates the implementation of the *Programa para la Optimización del Uso de Antimicrobianos* (PROA) in Chile in 2020.

When expressed relative to biomass, these differences become even more apparent and underscore structural contrasts in disease management and antimicrobial dependence among production systems. Whereas Norway and Canada have maintained low and stable levels of antibiotic use, Chile continues to show greater variability despite an overall tendency toward reduction. The results of this analysis are presented in Table 1 and Figure 4.

**Table 1:** Correlation analysis of temporal trends in antimicrobial use across major salmon-producing countries, including correlation coefficients and significance values.

Country	r	*p*_value
Canada	0.00004064327	0.99991720
Chile	−0.63700845631	0.06502783
Norway	0.02101163951	0.95721118

## 9 Future perspectives and research priorities: controlling SRS as the guiding principle for reducing antibiotic use

As part of its broader regulatory strategy, Chile has implemented a risk-based classification system for the management of piscirickettsiosis (SRS) (SERNAPESCA, 2012). Within this framework, salmon farms are placed under active surveillance through systematic PCR-based monitoring for *P. salmonis* (Gaete-Carrasco et al., 2019; SERNAPESCA, 2021). To ensure diagnostic reliability, sampling protocols require a minimum of 15 fish per event, preferably including stranded, moribund, or disoriented individuals, with liver, posterior kidney, and brain defined as target tissues (SERNAPESCA, 2021).

This classification system establishes distinct epidemiological risk categories linked to predefined regulatory actions. A farming site is designated as an “alert center” when any of the following criteria are met: (i) one or more cages register weekly mortality ≥0.35% attributable to *P. salmonis*; (ii) a cage receives three antimicrobial treatments within a three-month period; or (iii) the farm was previously classified as a high-dissemination center (in Spanish: Centro de Alta Diseminación, CAD). The CAD category is assigned when ≥50% of cages exceed the 0.35% mortality threshold or when elevated mortality persists for four consecutive weeks (SERNAPESCA, 2012). By directly linking mortality dynamics and treatment frequency to regulatory escalation, this risk-based system enables earlier intervention and aims to reduce reliance on antimicrobials (Price et al., 2016, 2020; Hillman et al., 2020). At all classification levels, a licensed veterinarian serves as the formal liaison with SERNAPESCA, ensuring compliance with health protocols and reporting obligations (SERNAPESCA, 2012, 2015).

**Figure 5:**
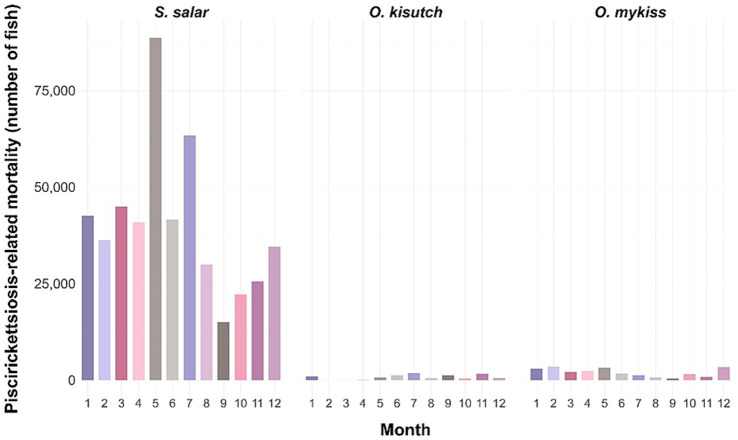
Monthly piscirickettsiosis-related mortality in Chilean salmon aquaculture. Bar plots show the total number of fish deaths attributed to piscirickettsiosis across all production centers, stratified by species: *Salmo salar*, *Oncorhynchus kisutch*, and *Oncorhynchus mykiss*. Values represent the cumulative number of individuals recorded each month for each species.

**Figure 6:**
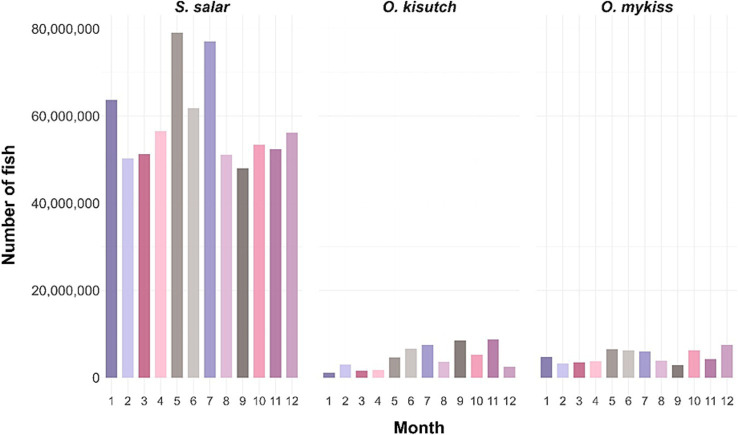
Monthly number of farmed salmonids exposed to metaphylactic antimicrobial treatments in Chile during 2023. Bar plots show the total number of individuals exposed each month, stratified by species: *Salmo salar*, *Oncorhynchus kisutch*, and *Oncorhynchus mykiss*. Values represent the cumulative number of farmed salmonids subjected to metaphylactic antibiotic treatment per month for each species.

Antimicrobial use associated with SRS control must be reported through SIFA, Chile’s centralized digital platform for monitoring antimicrobial prescriptions (Ministerio de Economía y SUBPESCA, 2014). SIFA compiles detailed information on antimicrobial class, dosage, duration, treated biomass, and treatment purpose, thereby providing a critical evidence base for policy evaluation and antimicrobial stewardship. Recent national data indicate that *S. salar* exhibits substantially higher SRS-associated mortality than *O. kisutch* and *O. mykiss* (Figure 5). In 2023 alone, an estimated 50–80 million *S. salar* reared at farms exceeding the 0.35% mortality threshold underwent metaphylactic antimicrobial treatment (Figure 6), underscoring the persistent disease pressure exerted by SRS (Hillman et al., 2020; SERNAPESCA, 2025).

**Figure 7:**
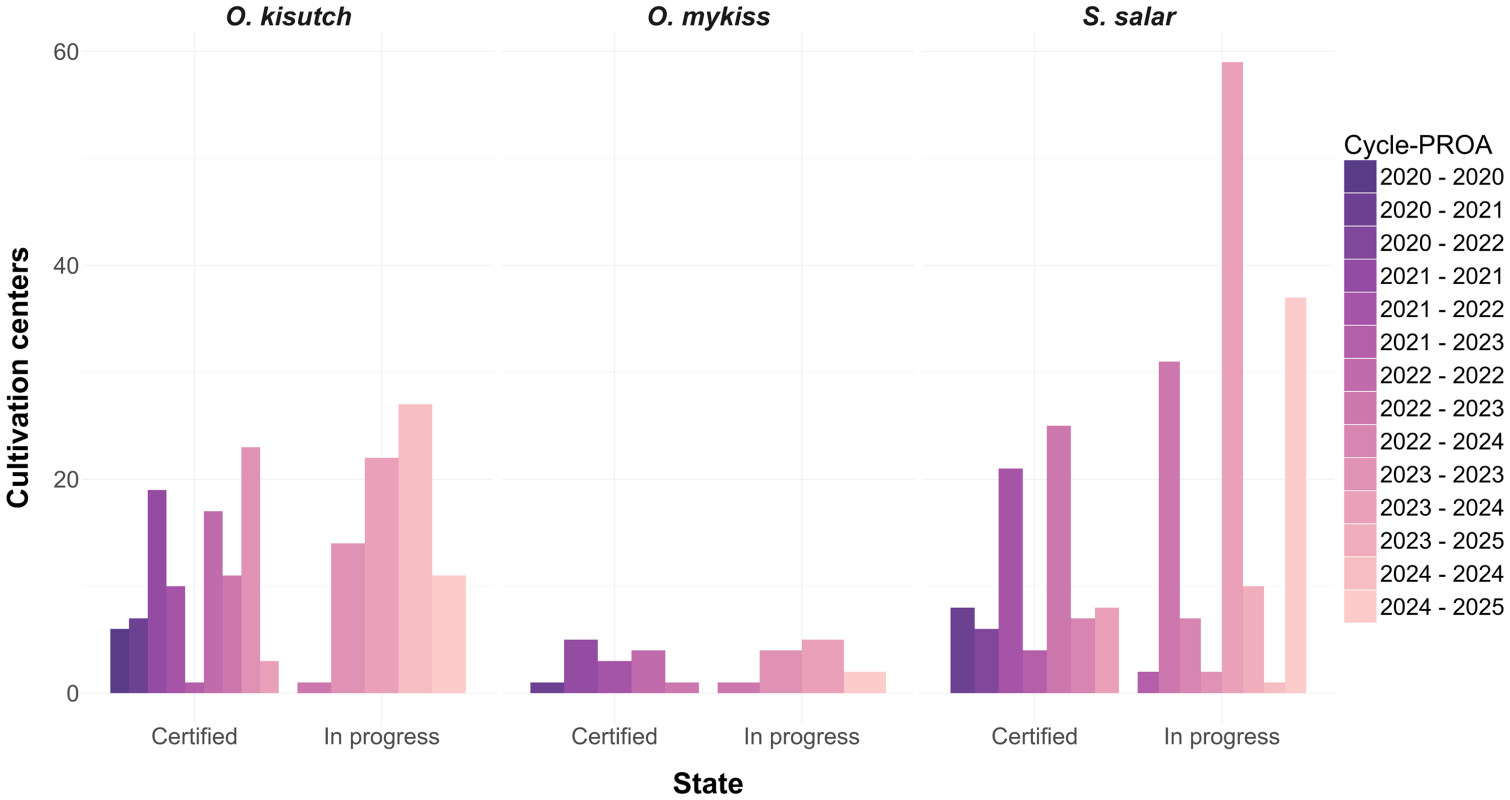
Progress of the Chilean Program for the Optimization of Antimicrobial Use (PROA) in salmon aquaculture. Bar plots show the number of salmonid farming centers, stratified by species (*Salmo salar*, *Oncorhynchus kisutch*, and *Oncorhynchus mykiss*) and grouped by certification status (certified vs. in progress). Colors indicate the production cycle (Cycle-PROA) during which each center applied for or obtained certification. Since its implementation, PROA has received more than 830 applications and has certified 287 production cycles.

**Figure 8:**
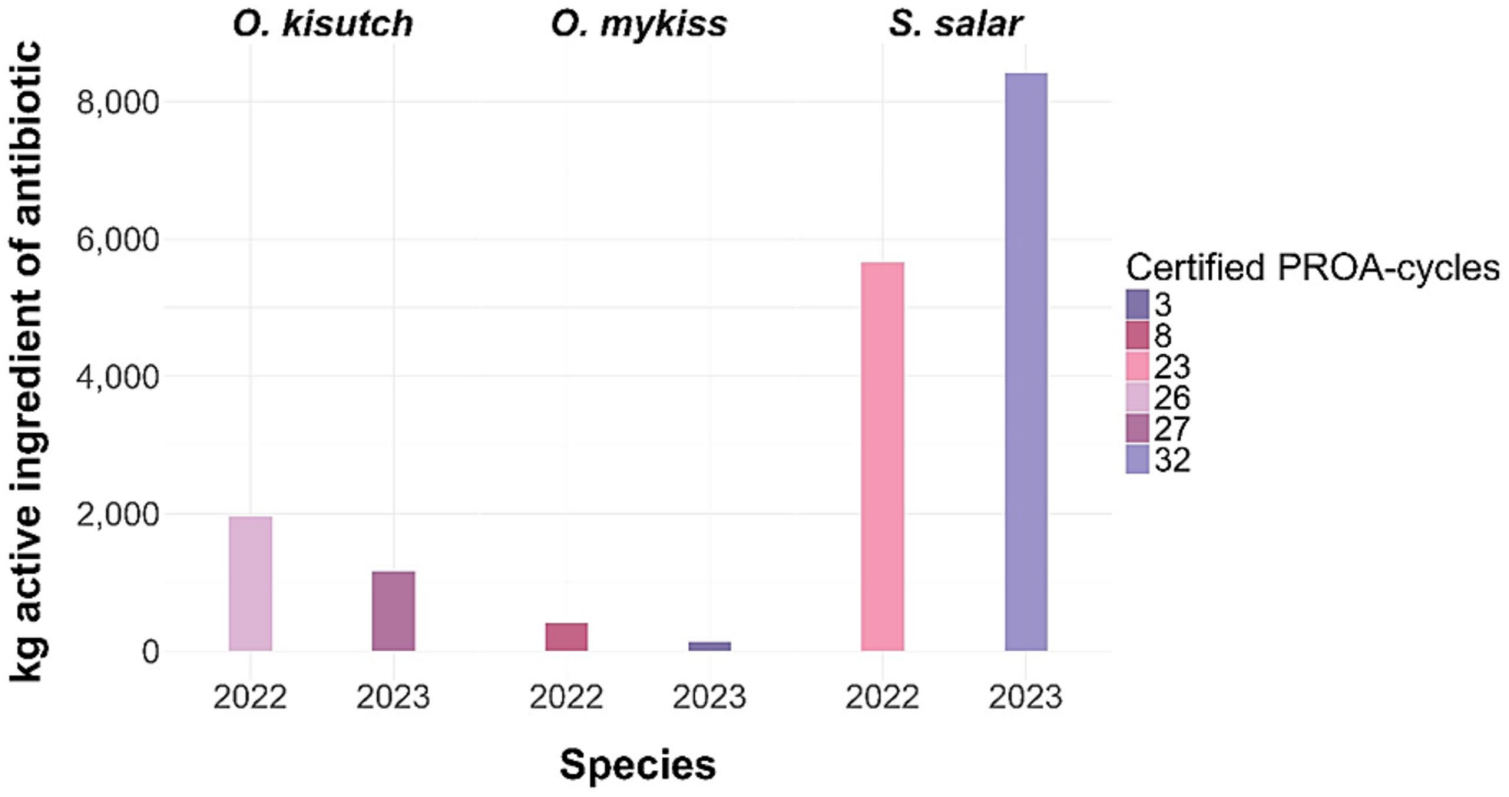
Certified PROA production cycles and associated antimicrobial use in Chilean salmonids during 2022–2023. Bar plots show the total kilograms of active antimicrobial ingredient used by species (*Oncorhynchus kisutch*, *Oncorhynchus mykiss*, and *Salmo salar*), with colors indicating the number of production cycles certified under the Program for the Optimization of Antimicrobial Use (PROA). The figure illustrates the distribution of certified cycles across species and years, highlighting the progressive adoption of PROA certification since its implementation in 2020.

To complement mandatory surveillance and regulatory oversight, Chile launched the Program for the Optimization of Antimicrobial Use (in Spanish: *Programa para la Optimización del Uso de Antimicrobianos*, PROA) in 2020. This voluntary certification scheme encourages producers to implement enhanced health-management strategies aimed at reducing antimicrobial dependence (SERNAPESCA, 2020). Since its inception, PROA has received more than 830 applications and has certified 287 production cycles (Figure 7). During the 2022–2023 period, PROA-certified farms harvested 397937.3 tons of salmon while using only 17734.25 kg of antimicrobials, with *S. salar* accounting for most of the certified production (Figure 8) (SERNAPESCA, 2020, 2025).

Certification under PROA requires implementation of a comprehensive health-management plan, including: (i) vaccination against SRS and other infectious diseases; (ii) prevention and control of sea lice infestations (*Caligus rogercresseyi*); (iii) PCR-based monitoring for *P. salmonis* at 15-day intervals; (iv) systematic staff training in mortality removal and necropsy procedures; and (v) the use of immunostimulants or alternative interventions aimed at delaying disease onset. In addition, participating farms must develop an Internal Plan for the Optimization of Antimicrobial Use (PROA), w|hich includes antimicrobial susceptibility testing of *P. salmonis* isolates using MIC-based methods for florfenicol and oxytetracycline (Yáñez et al., 2014; Henríquez et al., 2016; Contreras-Lynch et al., 2017; San Martín et al., 2019), prioritization of cage-level treatments, verification of medicated feed homogeneity, maintenance of adequate feed stocks, and strict adherence to withdrawal periods and registered conditions of use (Price et al., 2018).

Within the PROA framework, antimicrobial use per production cycle is capped at one oral or immersion treatment for *O. kisutch* and *O. mykiss*, and at a maximum of two treatments for *S. salar*, with upper use thresholds of 600 kg and 1,200 kg, respectively (excluding injectable formulations) (SERNAPESCA, 2020). Taken together, these restrictions, combined with enhanced surveillance and preventive measures, position SRS control as the central leverage point for achieving sustained reductions in antimicrobial use in Chilean salmon aquaculture.

## 10 Multiscale drivers of antimicrobial dependence in Chilean salmon aquaculture

Beyond pathogen prevalence and vaccine limitations, antimicrobial dependence in Chilean salmon aquaculture is also sustained by broader structural, biological, and environmental drivers. First, the industry operates within a highly export-oriented production model in which rapid growth, high stocking densities, and the need to maintain production continuity under sanitary pressure have historically favored intensive disease-control practices (Barton and Fløysand, 2010). Chilean salmon farming was described early as a major export business whose rapid expansion was accompanied by heavy antibiotic use, and subsequent reviews have confirmed that the country continues to display one of the highest antimicrobial consumption rates per ton of harvested salmon worldwide (Miranda et al., 2018; Farias et al., 2025). In this context, the economic pressure to avoid biomass losses, maintain harvest schedules, and protect product flow to international markets likely acts as an underlying incentive for continued reliance on antimicrobial interventions, particularly during periods of severe infectious pressure.

A second driver is the mismatch between the duration of the salmon production cycle and the persistence of disease susceptibility during the marine grow-out phase. Atlantic salmon production cycles can extend for approximately 36 months under commercial conditions, exposing fish to prolonged and changing environmental challenges across freshwater and seawater stages. This long production window creates repeated opportunities for stress-related deterioration of fish health and for the onset or recurrence of infectious disease. In parallel, piscirickettsiosis remains difficult to control, antibiotic treatment frequently fails, and currently available vaccines show variable long-term efficacy (Rozas and Enríquez, 2014; Coca et al., 2023). Together, these factors generate a temporal gap between the duration of production and the durability of effective protection, thereby favoring recurrent therapeutic or metaphylactic antimicrobial use (Valenzuela-Aviles et al., 2022).

A third driver involves host genetics. Resistance to *P. salmonis* shows significant genetic variation, and genomic prediction has been proposed as a feasible strategy to accelerate selection for improved resistance in Atlantic salmon (Bangera et al., 2017; Figueroa et al., 2020). At the same time, host genetic variation has been shown to influence vaccine-mediated protection, helping explain why commercial vaccines may perform inconsistently across families or populations (Valenzuela-Aviles et al., 2022). These findings indicate that breeding and selection schemes are not neutral with respect to antimicrobial use: when host resistance is suboptimal, farms may remain more dependent on antibiotics to compensate for incomplete preventive protection (Figueroa et al., 2020; Mukiibi et al., 2022). Conversely, better integration of genetic resistance into breeding programs could become an important long-term lever for reducing antimicrobial demand.

Environmental variability is another key multiscale driver of antimicrobial dependence in Chilean salmon aquaculture. Harmful algal blooms and low-oxygen conditions have become increasingly relevant stressors in southern Chile, where hydroclimatic anomalies, thermal stratification, and high organic loading can compromise fish health and destabilize production systems. The 2016 harmful algal bloom crisis is particularly illustrative: a bloom dominated by *Pseudochattonella cf. verruculosa* during the austral summer caused one of the most severe mortality events recorded in Chilean salmon farming, killing nearly 12% of national production and exposing the vulnerability of the industry to large-scale environmental disturbances (Mardones et al., 2021). In Atlantic salmon, hypoxic conditions have been associated with reduced appetite and growth, as well as altered stress- and immune-related responses, particularly when combined with elevated temperature, indicating that low oxygen can weaken physiological resilience under farming conditions (Burt et al., 2013). In parallel, recent analyses of piscirickettsiosis have identified harmful algal blooms and low-oxygen conditions as extrinsic stressors that may contribute to vaccine failure and increase host susceptibility during the seawater phase (Quiñones et al., 2019; Valenzuela-Aviles et al., 2022). In Chile, these environmental disturbances are especially important because they do not act in isolation: harmful algal blooms can generate or exacerbate oxygen depletion, and both processes may narrow the margin for preventive disease control, thereby reinforcing antimicrobial use as a short-term response to acute sanitary deterioration rather than as part of a preventive strategy (Quiñones et al., 2019; Beemelmanns et al., 2021; Valenzuela-Aviles et al., 2022).

**Figure 9:**
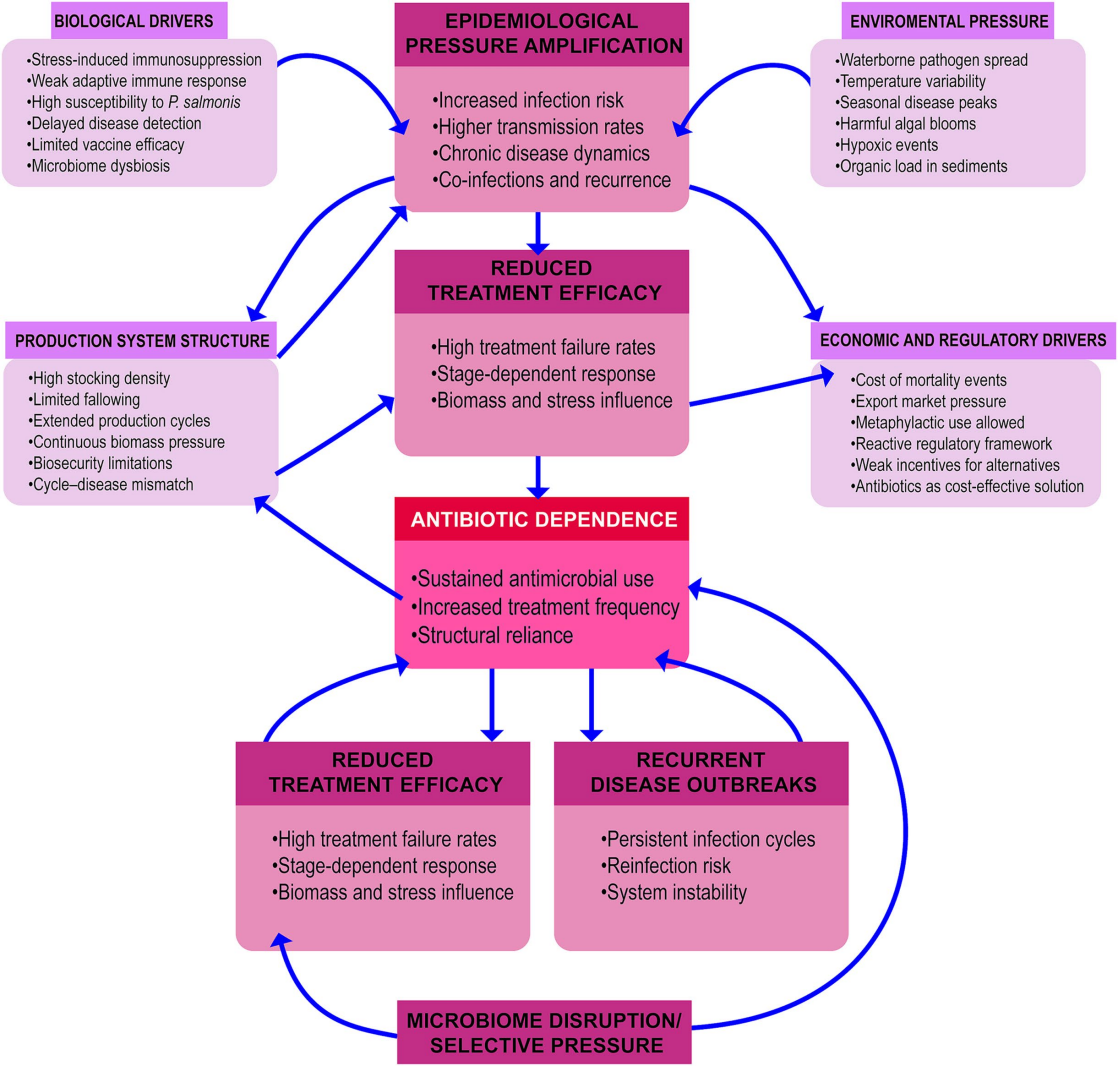
Systems-level drivers of antibiotic dependence in Chilean salmon aquaculture. Conceptual framework summarizing the multifactorial drivers that sustain antimicrobial dependence in intensive salmon production systems. Four interacting domains are shown: (i) Biological drivers, including host susceptibility to, limited vaccine efficacy, immunosuppression, and microbiome dysbiosis; (ii) environmental pressures, such as temperature variability, pathogen dispersion, harmful algal blooms, and hypoxic events; (iii) production-system design, including high stocking densities, limited fallowing, extended production cycles, and biosecurity constraints; and (iv) economic and regulatory forces, including export market pressures, the cost-efficiency of antibiotic use, and predominantly reactive regulatory frameworks.

Taken together, these drivers indicate that antimicrobial dependence in Chilean salmon aquaculture cannot be understood solely as a consequence of pathogen presence or insufficient regulation (Figure 9). Rather, it emerges from the interaction of export-oriented production pressures, long production cycles with prolonged exposure to disease risk, incomplete incorporation of host resistance into breeding strategies, and environmental stressors such as blooms and hypoxic conditions. Addressing this dependence therefore requires a broader preventive framework that integrates fish genetics, production design, environmental forecasting, and sanitary decision-making, rather than relying predominantly on antimicrobial intervention once disease has already become established.

## 11 Discussion and One Health implications

The Chilean salmon aquaculture industry exemplifies both the global importance of aquaculture as a food-production sector and the structural vulnerabilities inherent to intensive production systems. Its persistent reliance on antimicrobials, particularly florfenicol and oxytetracycline, reflects the central and still unresolved challenge posed by *P. salmonis*, a pathogen for which available vaccines remain insufficiently effective and alternative therapeutic options are limited (Rozas and Enríquez, 2014; San Martín et al., 2019; SERNAPESCA, 2025; Trincado et al., 2024). The contrast with Norway is particularly illustrative: despite comparable production volumes, Norway has achieved a > 99% reduction in antibiotic use through effective vaccination strategies, strict biosecurity, and tightly integrated regulatory frameworks (Lillehaug et al., 2018; Hoseinifar et al., 2024). In Chile, by contrast, disease control remains dominated by metaphylactic interventions, reflecting a combination of high SRS prevalence, limited vaccine performance, and constrained capacity for early preventive action.

The quantitative analysis of antimicrobial use trends further supports this interpretation. Although a decreasing tendency was observed in Chile (r = −0.64), this trend did not reach statistical significance (*p* = 0.065), indicating that reductions in antimicrobial use have not followed a sustained or consistent trajectory over time. Instead, the marked interannual variability suggests that antibiotic use patterns are likely driven by episodic disease dynamics, particularly those associated with *P. salmonis*, rather than by systematic improvements in health management. In contrast, the stable and consistently low levels of antibiotic use observed in Norway and Canada, where no significant temporal trends were detected (r = 0.02, *p* = 0.96; r ≈ 0.00, *p* = 0.9999, respectively), reflect production systems in which antimicrobial use is structurally controlled. These differences reinforce the view that, despite implementation of regulatory frameworks such as PGSA, PSEVC, and SIFA, antimicrobial use in Chile continues to respond to disease outbreaks rather than being effectively prevented through sustained, system-level strategies. At the same time, this dependence cannot be explained solely by pathogen burden or imperfect regulation. Rather, it emerges from the interaction of drivers operating across multiple biological, environmental, and production scales. At the production-system level, Chilean salmon aquaculture remains embedded in a highly export-oriented model in which harvest continuity, biomass preservation, and maintenance of production flow are economically critical. Within this context, sanitary deterioration can rapidly translate into substantial biological and commercial losses, favoring intensive disease-control practices during periods of severe infectious pressure. These structural pressures are compounded by the long duration of the production cycle relative to the persistence of disease susceptibility, particularly during the seawater phase, where fish remain exposed for prolonged periods to *P. salmonis*, environmental fluctuations, and cumulative physiological stress. Together, these features create a temporal and operational gap between the duration of production and the durability of effective protection, thereby reinforcing antimicrobial use as a recurrent risk-management tool.

Host-related factors also contribute to this pattern. Genetic resistance to *P. salmonis* is variable, and breeding schemes have increasingly been recognized as relevant to long-term disease control; however, the incorporation of host resistance into routine production remains incomplete. In parallel, the lack of standardized tools to evaluate protective immunity in teleosts means that antibody titers and immune correlates of protection remain poorly defined and difficult to operationalize in farm settings (Watts et al., 2001; Dixon, 2012; Ye et al., 2013). These limitations reflect broader knowledge gaps in teleost immunology and constrain the translation of vaccine performance and host resistance into evidence-based regulatory thresholds. As a result, when preventive protection is incomplete, antimicrobial treatments continue to function as compensatory tools within the production cycle.

Metaphylaxis, administered primarily through medicated feed, is operationally feasible at commercial scale but biologically inefficient. Diseased fish commonly exhibit reduced appetite, resulting in subtherapeutic exposure, whereas healthy or subclinically infected individuals, which sustain production, are unnecessarily medicated (Rozas and Enríquez, 2014; Price et al., 2019). This imbalance represents a critical operational gap, as it simultaneously undermines therapeutic efficacy and amplifies selective pressure for antimicrobial resistance (Andersson and Hughes, 2014). Importantly, this strategy largely overlooks the biological consequences of antimicrobial exposure in clinically healthy fish. Recent evidence demonstrates that florfenicol exposure, even at therapeutic and hypertherapeutic doses, alters the interaction between the intestinal microbiota and local immune responses, affecting a compartment central to nutrient absorption and host barrier function (Camacho-Méndez et al., 2025). These findings challenge the assumption that metaphylaxis is biologically neutral for non-diseased individuals and highlight an underappreciated cost of current control strategies.

Environmental variability adds a further layer of complexity. In southern Chile, harmful algal blooms, hydroclimatic anomalies, and low-oxygen conditions have become increasingly relevant stressors for salmon production. The 2016 harmful algal bloom crisis, which caused one of the most severe mortality events recorded in Chilean salmon farming, clearly illustrated how large-scale environmental disturbances can destabilize the production system and sharply reduce the margin for preventive disease control (León-Muñoz et al., 2018; Quiñones et al., 2019). Likewise, hypoxic conditions have been associated with reduced feed intake, impaired growth, and altered stress- and immune-related responses in Atlantic salmon, especially when combined with elevated temperature, suggesting that low oxygen can reduce host resilience under farming conditions (Burt et al., 2013; Beemelmanns et al., 2021). Recent analyses of piscirickettsiosis have further identified harmful algal blooms and low-oxygen conditions as extrinsic stressors that may contribute to vaccine failure and increase host susceptibility during the seawater phase (Valenzuela-Aviles et al., 2022). These processes are especially relevant because they do not act independently: blooms, oxygen depletion, high organic loads, and infectious pressure may converge to intensify sanitary instability and reinforce antimicrobial use as a short-term response to acute production risk.

At the regulatory level, antimicrobial decision-making remains heavily dependent on veterinary clinical judgment, in the absence of enforceable limits on cumulative dosing, the number of treatments per production cycle, or explicit consideration of immunological and ecological consequences in healthy fish. This reliance is compounded by the fact that current authorization schemes remain more closely tied to mortality thresholds and traceability requirements than to integrated biological indicators of host status, environmental stress, or preventive protection. In this sense, the Chilean system has advanced substantially in surveillance and accountability, but it still operates primarily as a response-oriented framework rather than as a preventive one.

Beyond farm-level inefficiencies, Chile’s antibiotic-intensive production model has well-documented environmental consequences. Numerous studies have consistently detected antibiotic-resistant bacteria and resistance genes in sediments, fish-associated microbiota, and surrounding waters, confirming that aquaculture environments act as reservoirs and dissemination pathways for antimicrobial resistance (Miranda and Zemelman, 2002; Buschmann et al., 2012; Shah et al., 2014; Higuera-Llantén et al., 2018; Ortiz-Severín et al., 2025). These observations reveal a disconnect between the ecological risks generated by repeated antimicrobial exposure and the mortality-based thresholds that currently guide sanitary management decisions. Environmental and microbiological impacts are rarely incorporated explicitly into the authorization criteria for successive treatments, despite their clear relevance within a One Health framework.

These findings must be interpreted within a broader One Health perspective, in which environmental, animal, and human health are interconnected through shared microbial and ecological networks. Although available evidence supports the existence of resistance reservoirs in aquaculture-impacted environments, the pathways linking these reservoirs to human clinical outcomes remain complex and only partially understood. This highlights the importance of distinguishing environmental connectivity from epidemiologically confirmed transmission, particularly when assessing the public health relevance of aquaculture-associated antimicrobial resistance.

Chile has nevertheless made significant progress in regulatory oversight and transparency. Instruments such as the Aquaculture Enforcement Information System (SIFA), the risk-based classification of farming centers, and the voluntary PROA certification program have strengthened accountability and created incentives to reduce antimicrobial use (SERNAPESCA, 2020, 2025). However, these mechanisms remain largely focused on traceability and *post hoc* reporting rather than prevention, reinforcing a predominantly reactive approach to disease control. By contrast, European systems integrate antimicrobial stewardship more closely with vaccine performance, early diagnostics, and surveillance-based intervention thresholds, enabling proactive alignment among host immunity, pathogen dynamics, and regulatory decision-making (NORM/NORM-VET 2019, 2019; Hoseinifar et al., 2024).

From a research and policy perspective, several critical gaps emerge. First, integrated surveillance frameworks that simultaneously address fish health, environmental resistomes, and public health indicators within shared geographic regions remain lacking. Second, genomic surveillance, including whole-genome sequencing and plasmid tracking, remains underutilized in Chile, limiting the ability to trace resistance determinants across ecological compartments and to distinguish clonal expansion from horizontal gene transfer. Third, current management strategies insufficiently incorporate microbiome dynamics, immunological status, host genetics, and ecological context into treatment authorization and disease-control planning. More broadly, the Chilean case suggests that antimicrobial dependence in intensive aquaculture must be understood as a multiscale phenomenon sustained by pathogen pressure, incomplete preventive protection, environmental instability, and production-system incentives acting simultaneously rather than independently.

Looking ahead, reducing antimicrobial dependence in Chilean salmon farming will require a paradigm shift toward integrated, preventive, and data-driven health management. Improving vaccine efficacy against *P. salmonis* remains essential (Maisey et al., 2017; Gaete-Carrasco et al., 2019), but this must be complemented by enhanced feed homogeneity to optimize drug delivery, cage-level diagnostics to enable targeted interventions, microbiome-informed strategies that preserve ecological balance, and immunological tools capable of informing regulatory thresholds (Camacho-Méndez et al., 2025). Strengthening biosecurity, husbandry practices, early-warning systems, environmental forecasting, and the incorporation of host resistance into breeding strategies will also be critical to reducing disease pressure before antimicrobial intervention becomes necessary.

Ultimately, the Chilean experience demonstrates that sustainable aquaculture cannot rely solely on ex post surveillance and reporting mechanisms. Instead, it requires preventive regulatory thresholds, biologically informed decision-making, and One Health-aligned risk assessment frameworks capable of reconciling production growth with environmental protection and public health (FAO, 2022; Cabello and Godfrey, 2023). Addressing Chile’s structural antibiotic dependence is therefore essential not only to preserve therapeutic efficacy and mitigate ecological and public health risks, but also to secure the long-term sustainability and international competitiveness of the salmon industry.

## 12 Conclusion

The Chilean salmon aquaculture industry provides a clear example of how intensive production systems can become structurally dependent on antimicrobials when effective preventive tools are lacking. Despite important advances in regulation, surveillance, and antimicrobial traceability, antibiotic use in Chile continues to be driven primarily by the persistent burden of salmon rickettsial syndrome (SRS) and the limited field efficacy of currently available vaccines against *Piscirickettsia salmonis*. This situation contrasts sharply with that of other major salmon-producing countries and highlights the inherent limitations of disease-control strategies that remain predominantly reactive rather than preventive.

At the same time, the Chilean case shows that antimicrobial dependence is not explained by pathogen burden alone. Rather, it is reinforced by multiscale drivers that include the structure of an export-oriented production model, the mismatch between long production cycles and prolonged disease susceptibility, incomplete integration of host resistance into preventive strategies, and the destabilizing effects of environmental stressors such as harmful algal blooms and low-oxygen conditions. These interacting drivers help explain why antibiotic use persists even in the presence of regulatory improvements and increasing surveillance capacity.

The predominance of metaphylactic treatments, largely administered through medicated feed, introduces major biological and operational inefficiencies, including heterogeneous drug exposure, unnecessary treatment of clinically healthy fish, and sustained selective pressure on both host-associated and environmental microbial communities. These dynamics not only compromise therapeutic precision but also favor the maintenance and dissemination of antimicrobial resistance within aquaculture-associated microbiota and surrounding aquatic environments, reinforcing the need to understand salmon farming as a One Health issue rather than solely as a production challenge.

Although acquired resistance in primary fish pathogens remains comparatively limited, the evidence reviewed here emphasizes the broader ecological and public health significance of resistance circulating in opportunistic bacteria and environmental reservoirs. In this context, the Chilean case shows that the most important consequences of antibiotic-intensive aquaculture may extend beyond treatment failure in target pathogens, involving the enrichment, persistence, and potential spread of resistance determinants across interconnected animal, environmental, and human compartments. Current surveillance systems are robust in terms of reporting and traceability, but they remain insufficiently integrated to capture these resistance flows in a coordinated and preventive manner.

Reducing antimicrobial dependence in Chilean salmon aquaculture will require a transition toward preventive, biologically informed, and data-driven health management. Key priorities include improving vaccine performance against SRS, strengthening early diagnostics and targeted intervention strategies, expanding integrated genomic, microbiological, and environmental surveillance to better inform antimicrobial stewardship, and incorporating host genetics, environmental forecasting, and ecological risk into routine disease-control frameworks. Advancing these approaches will be essential to preserve therapeutic efficacy, protect aquatic ecosystems, and ensure the long-term sustainability, legitimacy, and international competitiveness of the Chilean salmon industry.
